# Development and Validation of an Atrial Fibrillation Knowledge Assessment Tool (AFKAT)

**DOI:** 10.3390/ijerph17051721

**Published:** 2020-03-06

**Authors:** Ibrahim Jatau Abubakar, Barbara C. Wimmer, Luke R. Bereznicki, Corinna Dwan, J. Andrew Black, Woldesellassie M. Bezabhe, Gregory M. Peterson

**Affiliations:** 1 School of Pharmacy and Pharmacology, College of Health and Medicine, University of Tasmania, Private Bag 26, Hobart, TAS 7001, Australia; barbara.wimmer@utas.edu.au (B.C.W.); luke.bereznicki@utas.edu.au (L.R.B.); corinna.dwan@utas.edu.au (C.D.); woldesellassie.bezabhe@utas.edu.au (W.M.B.); g.peterson@utas.edu.au (G.M.P.); 2 Royal Hobart Hospital, Hobart, TAS 7000, Australia; andrew.black@ths.tas.gov.au

**Keywords:** atrial fibrillation, knowledge, assessment, survey, validation

## Abstract

Assessing and improving public knowledge of atrial fibrillation (AF) could increase its detection rate and the subsequent use of stroke prevention therapies. However, there is no validated AF knowledge assessment tool applicable to the general population, including those at risk of AF. Therefore, we aimed to develop and validate such a tool. The tool was developed from a literature review and discussion with subject matter experts. Content validity was ensured by a ten-member panel of experts comprising cardiologists and pharmacists. An online validation survey was conducted and reported based on the Checklist for Reporting Results of Internet E-Surveys (CHERRIES). The survey evaluated the tool performance by construct validity, internal consistency reliability, item discrimination, difficulty index and ease of readability. The survey participants included 14 general medical specialists, 20 fourth-year and 33 second-year undergraduate pharmacy students, and 122 members of the general public. The tool had satisfactory content validity, with a scale content validity index of 0.8. The mean percentage knowledge scores for general medical specialists and fourth-year pharmacy students were higher than second-year pharmacy students, followed by the general public (92.9%, 87.6%, 68.5% and 53.4%, respectively; *p*-value < 0.001), supporting construct validity. The tool had good internal consistency reliability (Cronbach’s alpha = 0.91). The item-total correlation was in the preferred range of 0.23 to 0.71. The Atrial Fibrillation Knowledge Assessment Tool is a valid instrument and can be used to investigate AF knowledge of the general population.

## 1. Introduction

Early detection and subsequent treatment of atrial fibrillation (AF) are crucial in preventing AF-related complications, including stroke [1]. Treatment can be delayed when AF is not detected early [2] and untreated AF, even in the absence of symptoms, can result in a stroke [3]. Poor knowledge of AF in the general population can potentially lead to under-detection and treatment delay. 

The literature suggests that approximately 30% of older people are not able to identify signs of AF, and nearly 50% are not aware of AF as a medical condition [2,4]. Patients with a new diagnosis of AF may have limited knowledge regarding AF symptoms [5,6], and approximately 40% of these patients report being unaware of AF prior to their diagnosis [7]. These knowledge and awareness deficits about AF suggest a need for a thorough assessment of AF knowledge of the general population. 

Poor knowledge of disease in the general population has been recognised as a significant barrier to the uptake of screening programmes in the community [8,9]. People with poor knowledge of AF may be less inclined to participate in AF screening programmes. Therefore, improving AF knowledge through educational interventions may motivate people to participate in screening programmes and seek early treatment for AF. To evaluate the impact of such interventions, a psychometrically validated tool is necessary to establish the baseline level and measure changes in AF knowledge. However, there is an absence of such a tool in the literature. 

Although some studies have evaluated the impact of educational programmes on improving AF knowledge of the general public [10] and individuals at high risk of AF [11], none of these studies utilised a psychometrically validated tool. These limitations highlight the need for a psychometrically validated tool that would be suitable for assessing knowledge of AF in a range of populations. To address this gap, we aimed to develop and validate such a tool. 

## 2. Materials and Methods

### 2.1. Development of AF Knowledge Assessment Tool

Items were generated from a review of the literature on AF knowledge retrieved from PubMed, Scopus, Embase and CINHAL databases. Studies were searched from inception of the databases to July 2018, using the following keywords: atrial fibrillation, knowledge, education, and awareness. Due to limited research on AF knowledge and to ensure comprehensiveness, further potential items were obtained through discussions with experts on the subject matter. Disagreements between authors regarding study selection and item inclusion were resolved through discussion until consensus was reached. The AF knowledge scope covered by this tool included basic AF information, risk factors, detection, prevention, and management. The format of the items was based on the guideline for developing questionnaires by Boateng et al. [12]. For each item, a participant could select a response out of three options (i.e., “True”, “False” or “I don’t know”). A correct response was given a score of ‘1’ and an incorrect or ‘I don’t know’ was given a score of ‘0’.

### 2.2. Psychometric Testing of the Tool

#### Face Validity

To assess face validity, an online format of the draft tool was developed and presented to a convenience sample of 20 people from the general public (10 each from medical and non-medical backgrounds). The link to the draft tool was shared with the participants via social media accounts (Facebook and WhatsApp). They were asked to respond to the items in the tool and to comment on the ease of understanding, and to identify terms that seemed ambiguous. Participants made comments in a text box provided after each item in the tool.

### 2.3. Content Validity 

To ensure all items in the draft tool were relevant for the study purpose, a ten-member expert panel consisting of three hospital cardiologists and seven academic pharmacists was constituted. The pharmacists were recruited from the University of Tasmania and the cardiologists from the Royal Hobart Hospital (RHH), the largest public hospital in Tasmania. These experts were selected based on their clinical and research experience. The experts were asked to rate the relevance of each item in the tool using a 4-point Likert scale (1 = not relevant, 2 = somewhat relevant, 3 = relevant, 4 = very relevant), and to suggest other items for the tool which may not have been considered. 

The content validity index of each item (I-CVI) was calculated as suggested by Polit and Beck [13], by dividing the number of experts who rated an item as relevant and very relevant (3 and 4) on the 4-point scale by the total number of experts [13]. The CVI of the scale (S-CVI) was determined using the Universal Agreement (UA) approach. The scale (S-CVI/UA) was computed by taking the overall mean CVI of items where the CVI was ≥0.70 [13]. I-CVI values of >0.78 and an S-CVI/UA value of ≥0.8 were considered acceptable based on recommendations from previous studies [13,14].

In addition to CVI, we used the Wynd et al. [15] recommendation in calculating a modified multi-rater kappa index (k*) for each item to adjust for chance agreement and provide an index of the degree of agreement among experts [15]. The k* was calculated using the formula; k* = (I-CVI − pc)/(1 − pc), where pc is the probability of a chance occurrence [16]. The pc was determined using binomial random variable probabilities in SPSS version 24 [17]. A k* value of 0.74 to 1.0 was considered excellent based on recommendations from previous studies [15]. 

### 2.4. Validation Study

To test other psychometric properties of the tool (construct validity, reliability, readability and difficulty index), a validation study was conducted among four sub-groups of people with different educational backgrounds and exposure to medical practice. The study groups included fourth- and second-year undergraduate pharmacy students (who had varying curriculum exposure to AF), general medical specialists, and people from the general public (aged ≥40 years, in whom the risk of developing AF starts to increase [18]). 

There was no previous data (from a pilot study) available to determine the statistical power analysis for the sample size calculation. Therefore, we estimated that the participants from the general public would score a mean percentage ± standard deviation (SD) of 50% ± (20) with the AF knowledge tool, with a difference in the mean score of 10% between sub-groups. Assuming alpha = 0.05 and power = 0.8, we calculated a minimum sample size of 48 or 12 participants per sub-group using G*Power sample size calculator [19]. The items used for the validation study are shown in Appendix A.

### 2.5. Recruitment of Participants

The general medical specialists were recruited from the RHH. Based on convenience, undergraduate pharmacy students from the University of Tasmania were recruited through second- and fourth-year class coordinators. The recruitment of participants from the public was done using a Facebook advertisement. Participants from the general public and undergraduate pharmacy students were excluded if they had been diagnosed with AF or were registered health care practitioners. A $10 gift voucher was used as an incentive to encourage the participation of members from the general public and the students.

### 2.6. Study Procedures

The validation study was a cross-sectional design in the form of an online survey (using LimeSurvey). The online survey was designed and reported based on the Checklist for Reporting Results of Internet E-Surveys (CHERRIES) [20]. All items in the tool were made mandatory for respondents and presented on one LimeSurvey page. Additionally, participants could review and change their responses through a back button on the page. To prevent multiple entries from the same participant, the Internet Protocol (IP) address of the client device was used to identify potential duplicate entries from the same user. Access to the survey tool was available through a link, where the information about the study and consent were made available before participants could proceed to the actual survey content.

The link to the LimeSurvey page was shared with the general medical specialists and the students via their institutional email addresses. Members of the general public could access the LimeSurvey page by clicking on the Facebook advert. The completion rate of the survey was calculated by dividing the number of participants who finished the survey (complete responses) by the total number of participants who responded to the survey (complete and incomplete responses) [20]. The mean time to complete the survey by the participants from the general public was obtained from the timestamp of the survey responses. A sub-analysis (not reported in this study) was conducted to ensure that all the logged completion times were sufficient for the participants to read, understand and answer the questions.

### 2.7. Construct Validity 

Construct validity was determined using a contrast group approach by comparing the mean knowledge scores of the four study sub-groups [21]. We hypothesised that the knowledge score of general medical specialists would be highest, followed by pharmacy students, and then the general public.

### 2.8. Internal Consistency Reliability

Cronbach’s alpha was used to determine the internal consistency reliability of the total responses. An alpha value of 0.70 was considered as sufficient and acceptable for a new scale [21]. The unidimensionality of the tool was assessed using the mean inter-item correlation [22]. A mean inter-item correlation value of 0.20 to 0.40 was considered acceptable [23].

### 2.9. Item Discrimination

Item discrimination was determined using the corrected item-total correlation. This test measures how an item correlates with the total score [24]. A correlation value of less than 0.2 was used as the cut-off value below which an item should be considered redundant and removed from the tool, as recommended by Streiner et al. [24]. 

### 2.10. Difficulty Index

Items that appeared to be ambiguous or difficult to understand by the respondents were identified using a difficulty index. It was calculated by dividing the number of correct responses by the total number of responses [25]. The higher the value, the easier the item is to be correctly answered. An item with a difficulty index of less than 50% was considered difficult to answer by the respondents [25].

### 2.11. Readability Analysis

To ensure ease of readability for the majority of the general population, the final draft AF public knowledge assessment tool was assessed using a Simplified Measure of Gobbledygook (SMOG) grade level [26]. The SMOG grade was determined using an online application called Reader.io [27]. A reading grade level of 8 to 9 is considered acceptable for health information in Australia [28]. 

### 2.12. Statistical Analysis

Statistical analysis was performed using IBM SPSS Statistics for Windows, Version 25.0. Armonk, NY: IBM Corp. Completed responses were exported from LimeSurvey to the SPSS software. Data was screened for potential meaningless responses prior to the analysis. AF knowledge scores of the study sub-groups were presented as mean percentages (SD). The difference in knowledge scores between the study sub-groups was analysed using one-way analysis of variance (ANOVA) and Tamhane’s T_2_ post hoc test. The Tamhane’s T_2_ was used in the post hoc test because an equal variance was not assumed among the study sub-groups [29]. A *p*-value of 0.05 was considered statistically significant.

Ethical approval: The study protocol was reviewed and approved by the Tasmanian Health and Medical Human Research Ethics Committee, reference number H0017308. All responses to the survey were anonymous. 

## 3. Results

**Figure 1:**
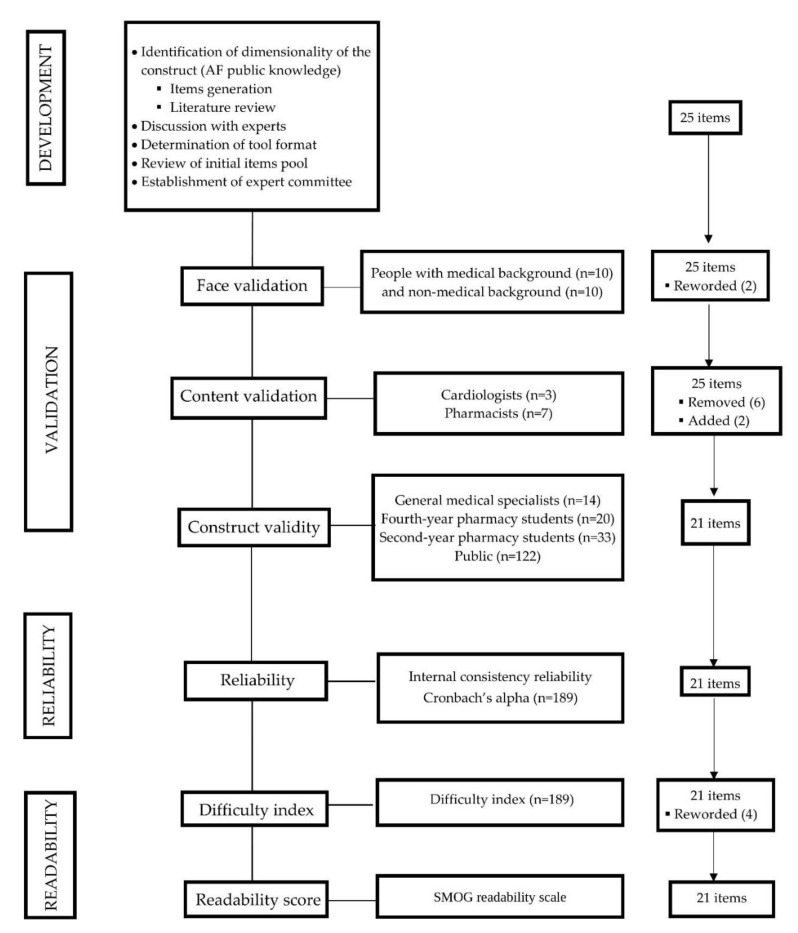
An outline of the development and validation process of the atrial fibrillation knowledge assessment tool (AFKAT). SMOG: Simplified Measure of Gobbledygook.

The draft AF public knowledge assessment tool consisting of 25 close-ended items was generated from the review of literature and discussion with experts on the subject matter. Figure 1 illustrates the outline of the development and validation processes of the AF knowledge assessment tool (AFKAT).

### 3.1. Face Validity

The participants judged the items in the draft tool as being easy to understand in terms of wording, response format and instructions. Two items were reworded based on the feedback received.

### 3.2. Content Validity Index

The S-CVI/UA value of the tool was 0.8, and the modified kappa value of 19 items were within a range of 0.7 to 1.0. Fifteen items had an I-CVI of more than 0.78 and were retained, while ten items with an I-CVI value of less than 0.78 were assessed by the research team for removal from the tool. Of the ten items, four of five items with an I-CVI value of 0.7 were retained as they were considered to be related to core information about AF. Two of the four items retained were reworded before inclusion. Appendix A shows the I-CVI and modified kappa values of the items in the tool.

Finally, two new items were suggested by the panel for inclusion in the tool. After a review, the items were considered relevant by the authors and included to form the tool consisting of 21 items (Table 1).

### 3.3. Validation Study

A total of 287 participants (members of the general public (166), second-year pharmacy students (60), fourth-year pharmacy students (39), and general medical specialists (22)) commenced the online survey. Accordingly, 122 (73.5%), 33 (55.0%), 20 (51.3%), and 14 (63.6%) of the responses from the general public, second-year pharmacy students, fourth-year pharmacy students, and general medical specialists, respectively, were fully completed. A total of 189 complete responses was therefore included in the final analysis, with an overall completion rate of 65.9%. The mean time taken for the general public participants to complete the survey was two and a half minutes. The results of the sub-analysis (not reported in this study) indicates that the completion time of two and half minutes should be sufficient for participants from the general public to read, understand and answer the survey items.

### 3.4. Construct Validity

The mean percentage (SD) of the knowledge score among the respondents is presented in Table 2. The ANOVA test showed a statistically significant difference in mean AF knowledge score across the four study sub-groups (*p*-value < 0.001). After post hoc analysis, the mean percentage knowledge scores of both the general medical specialists and the fourth-year pharmacy students was found to be significantly higher than that of the second-year pharmacy students and the general public sub-groups (*p*-value < 0.001). The mean knowledge score of the second-year pharmacy student sub-group was significantly higher than that of the general public sub-group (*p*-value = 0.005). There was no statistically significant difference between the general medical specialists’ sub-group and the fourth-year pharmacy students’ sub-group (*p*-value = 0.238).

### 3.5. Internal Consistency-Reliability

For the total number of participants (*n* = 189), a Cronbach’s alpha value of 0.91 was determined. A Cronbach’s alpha value of 0.90 was obtained for the participants from the general public, 0.83 for second-year pharmacy students, 0.32 for fourth-year pharmacy students, and 0.48 for general medicine specialists. The removal of any single item from the tool was not found to significantly increase the alpha value of the tool overall. The changes in the Cronbach’s alpha value for all participants if an item was deleted are shown in Table 3. The mean inter-item correlation of the tool was found to be 0.33.

### 3.6. Item Discrimination

The value of the corrected item-total correlation with the scale overall was observed to be in the range of 0.23 to 0.71 (Table 3). 

### 3.7. Difficulty Index

The difficulty index of the items ranged from 30.2% to 82.5% (Table 3). Four items were found to have a difficulty index of below 50% and were considered ambiguous. These items were further reviewed and reworded. 

### 3.8. Readability Index

The SMOG grade level of the tool was found to be 13.5. The grade level reduced to 9.2 when the term “atrial fibrillation” was removed. However, the term was retained based on its relevance to the study purpose. The final 21-item AFKAT is provided in Table 1.

## 4. Discussion

In this study, we described the development and validation of a tool for assessing AF knowledge of the general public. The results of extensive psychometric testing and the mean time taken for the general public participants to complete the survey (two and a half minutes) suggest that the AFKAT is easy to understand and to administer. 

A previous study measured older people’s knowledge, attitudes, and beliefs regarding AF using a scale where the content validity and internal consistency reliability were assessed [2]. However, this tool has some limitations that may affect its suitability for assessing the AF knowledge of the general public. Firstly, other psychometric properties such as readability and difficulty index, and sensitivity of the scale in measuring AF knowledge were not reported. Secondly, the mode of administration was interviewer-guided, which is more time consuming and costly in comparison to self-administration [30]. The tool’s third limitation was its relatively narrow scope, which captured only limited components of AF knowledge. Unlike this scale [2], our tool assesses AF knowledge as a single outcome measure, covers a broad spectrum of AF knowledge, and it appeared to have good psychometric features that would enable self-administration by a wide range of populations.

The methods applied in this study were based on the recommendations of previous studies for testing psychometric properties of a scale [12,13,21,24]. For the determination of face validity of a tool, two opinions exist in the literature regarding the inclusion of either laypersons or people with background knowledge of the subject matter [31]. In this study, both people with and without background knowledge of the subject matter were included.

For content validation of a tool, several methods have been suggested in the literature [21]. The CVI was used in this study because it is easy to calculate and understand, it focuses on agreement based on relevance, and consensus rather than consistency, and it provides both items- and scale-level information [32]. The S-CVI/UA value of the tool was within the acceptable range, suggesting that the tool had relevant content that can measure AF knowledge of the public [14]. Despite its advantages, the CVI has a limitation of failure to adjust for a chance of agreement [32]. This weakness was addressed in this study by computing the modified kappa of each item in the tool, and values corresponding to either good or excellent strength of agreement were obtained. 

The developed tool supported the principles of hypothesis testing and contrast group approach for construct validity [21]. We observed that there was no statistically significant difference in mean AF knowledge scores between the general medical specialists’ sub-group and the fourth-year pharmacy students’ sub-group. This finding reflects a ceiling effect whereby a reasonable knowledge of clinical medicine results in a near-perfect score with a tool intended for use in the general public. 

We used Cronbach’s alpha to measure the internal consistency reliability of the tool because of its ability to be applied with one test administration [21]. Opinion differs about the acceptable alpha value. Some experts recommend an ideal alpha value of 0.90 [33], while others suggest a value of 0.70 for a new scale [21]. The overall alpha value of our tool (0.91) was consistent with the two recommendations. The value of Cronbach’s alpha was not consistent among the groups, most likely reflecting the intended purpose and audience of the AF knowledge tool; critically, the alpha value for participants from the general public (0.90) was high.

The tool had a satisfactory difficulty index, with 19 out of the 21 items having an index of 51% to 84%, corresponding to “moderate” difficulty [25]. This finding implies that the tool was easily understood and could be self-administered by most participants with little difficulty. The SMOG grade level was used to assess the ease of readability of the tool [26]. This approach is considered to be the gold standard for assessing health information, and it is recommended by the United States Institutes of Health [34]. 

The present study has the following public health implications. Firstly, the validation study has demonstrated AFKAT’s potential usefulness in assessing the AF knowledge of the general public. Secondly, the AFKAT has recently been used in a large-scale, population-based Australian sample using an online survey, in a separate study by the authors. That study aims to assess the baseline AF knowledge of the Australian population and subsequently measure the impact of educational interventions. The AFKAT appears to be useful in assessing the baseline knowledge of AF and evaluating the effectiveness of educational interventions. Thirdly, this tool may be used in other studies aimed at exploring AF knowledge of the general public. Therefore, it would be desirable to develop versions in a wide range of languages and available for use in different populations. It could be utilised in the future by healthcare providers and relevant organisations to (i) examine knowledge gaps in the adult population and individuals at risk of AF; (ii) guide the development of AF information materials; and (iii) evaluate the impact of educational interventions for improving AF knowledge in the community.

This study has several potential limitations. The online nature of the survey for the recruitment of participants from the general population may have restricted respondents to those with internet access. Such individuals may have had more education and access to health-related information. However, the mean knowledge score was still significantly lower than the student and general medical specialist sub-groups. Being an online un-supervised quiz means that indviduals could have accessed the answers from available resources. 

## 5. Conclusions

The atrial fibrillation knowledge assessment tool is a valid instrument and can be used to investigate AF knowledge of the general population. 

## Figures and Tables

**Table 1:** AF knowledge assessment tool (AFKAT).

S/N	Item	Correct Answer
1	Atrial fibrillation is a medical condition where the heart beats slower than normal.	False
2	Atrial fibrillation may cause blood clots in the heart.	True
3	Episodes of atrial fibrillation are predictable.	False
4	People with atrial fibrillation can still have an active life.	True
5	Atrial fibrillation can only be treated with surgery.	False
6	Episodes of atrial fibrillation can be recurrent.	True
7	Early diagnosis and management of atrial fibrillation can prevent stroke.	True
8	Low blood pressure increases the risk of developing atrial fibrillation.	False
9	Atrial fibrillation significantly increases the risk of stroke.	True
10	Atrial fibrillation occurs only in people with prior signs of heart disease.	False
11	Shortness of breath and fainting can be potential symptoms of atrial fibrillation.	True
12	Atrial fibrillation occurs only in old age.	False
13	Someone could have atrial fibrillation without having any symptoms.	True
14	Symptoms of atrial fibrillation may be occasional, persistent, or permanent.	True
15	Atrial fibrillation usually has major psychological effects on people’s lives.	False
16	The risk of developing atrial fibrillation can be reduced with lifestyle changes.	True
17	Atrial fibrillation can be detected by checking the regularity of the pulse.	True
18	Screening for atrial fibrillation is safe.	True
19	Once present, atrial fibrillation is always a lifelong condition.	False
20	Atrial fibrillation can be treated with medications.	True
21	Anticoagulants (“blood thinners”) are often used to reduce the risk of stroke in people with atrial fibrillation.	True

**Table 2:** Mean percentage of AF knowledge scores of the study sub-groups.

Study Sub-Groups	Mean Percentage Score (SD)	F Statistics (df)	*p*-Value *
General medical specialist (*n* = 14)	92.9 (6.9)	21.044 (3, 185)	<0.001
Fourth-year pharmacy student (*n* = 20)	87.6 (7.5)		
Second-year pharmacy student (*n* = 33)	68.5 (20.4)		
Members of the general public (*n* = 122)	53.4 (27.7)		

* One-way ANOVA.

**Table 3:** Psychometric properties of the AFKAT by item.

Item Number	Difficulty Index	Item-Total Correlation	Cronbach’s Alpha *
1	55.6	0.54	0.91
2	57.7	0.39	0.91
3	82.5	0.66	0.90
4	30.2	0.39	0.91
5	60.3	0.64	0.90
6	79.4	0.71	0.90
7	79.9	0.69	0.90
8	67.7	0.58	0.90
9	68.3	0.63	0.90
10	37.0	0.48	0.91
11	64.6	0.67	0.90
12	76.7	0.66	0.90
13	66.7	0.52	0.91
14	73.5	0.64	0.90
15	67.7	0.38	0.91
16	46.6	0.23	0.91
17	42.3	0.32	0.91
18	66.7	0.46	0.91
19	68.8	0.67	0.90
20	58.7	0.63	0.90
21	64.0	0.63	0.90

* Cronbach’s alpha if an item was deleted.

## Appendix A

**Table A1:** Content validity and modified kappa of the individual items in the AFKAT.

S/N	Item	I-CVI	Modified Kappa (k*) $k*=\frac{I-CVI-pc}{1-pc}$
1	Atrial fibrillation is a medical condition where the heart beats slower than normal.	0.8	0.8
2	The precise cause of atrial fibrillation is unknown.	0.3	0.2
3	Atrial fibrillation occurs only in old age.	0.8	0.8
4	Atrial fibrillation is a lifelong condition.	0.9	0.9
5	Atrial fibrillation may cause blood clots in the heart.	0.8	0.7
6	People with atrial fibrillation can still have an active life.	0.9	0.9
7	Episodes of atrial fibrillation can be recurrent.	0.9	0.9
8	Episodes of atrial fibrillation are predictable.	0.8	0.7
9	Atrial fibrillation is diagnosed using a chest X-ray.	0.3	0.18
10	Early diagnosis of atrial fibrillation can prevent stroke.	0.9	0.9
11	Hypertension increases the risk of developing atrial fibrillation.	0.9	0.9
12	Atrial fibrillation significantly increases the risk of stroke.	1.0	1.0
13	Atrial fibrillation is associated with a heart attack.	0.6	0.5
14	Atrial fibrillation, by itself, is life-threatening.	0.30	0.2
15	Shortness of breath, chest pain can be potential symptoms of atrial fibrillation.	0.7	0.7
16	Someone could have atrial fibrillation without having any symptoms.	1.0	1.0
17	Symptoms of atrial fibrillation may be occasional, persistent, or permanent.	0.9	0.9
18	Atrial fibrillation has psychological effects on people’s lives.	0.8	0.9
19	Atrial fibrillation is modifiable with lifestyle changes.	0.9	1.0
20	Avoiding excessive alcohol intake can prevent atrial fibrillation.	0.7	0.7
21	Atrial fibrillation can be detected by checking the pulse rate.	0.8	0.8
22	Screening for atrial fibrillation may reduce the risk of developing a stroke.	0.5	0.3
23	Atrial fibrillation can be treated with medications.	0.8	0.8
24	Atrial fibrillation can be treated with surgery.	0.7	0.7
25	The goal of treating atrial fibrillation is to increase the heart rate.	0.3	0.2

Note: pc (probability of a chance occurrence) was calculated using binomial random variable probabilities in SPSS version 24 [17]. I-CVI is the item content validity index; k* = kappa indicating the strength of agreement on relevance. k* value of < 0.40 (poor), 0.40 to 0.59 (fair), 0.60 to 0.74 (good), and 0.74 to 1.00 (excellent). Scale content validity index for items with I-CVI ≥0.70 (S-CVI/UA) = 0.8.
